# A generational comparison for unfavorable cancer of unknown primary in a single institute over 20 years

**DOI:** 10.1002/cam4.4960

**Published:** 2022-07-03

**Authors:** Cheng‐Yu Tang, Jen‐Fan Hang, Yi‐Ping Hung, Nai‐Chi Chiu, Jiun‐I Lai, Ming‐Huang Chen, Chun‐Yu Liu, Muh‐Hwa Yang, Yee Chao, Peter Mu‐Hsin Chang

**Affiliations:** ^1^ Division of Medical Oncology, Department of Oncology Taipei Veterans General Hospital Taipei Taiwan; ^2^ School of Medicine National Yang Ming Chiao Tung University Taipei Taiwan; ^3^ Department of Pathology and Laboratory Medicine Taipei Veterans General Hospital Taipei Taiwan; ^4^ Department of Radiology Taipei Veterans General Hospital Taipei Taiwan; ^5^ Institute of Clinical Medicine National Yang Ming Chiao Tung University Taipei Taiwan; ^6^ Genomics Research Center, Academia Sinica Taipei Taiwan; ^7^ Genome Research Center National Yang‐Ming Chiao Tung University Taipei Taiwan; ^8^ Institute of Biopharmaceutical Sciences National Yang Ming Chiao Tung University Taipei Taiwan

**Keywords:** C‐reactive protein, performance status, prognosis, unknown primary tumors

## Abstract

**Background:**

The prognosis of unfavorable cancer of unknown primary is extremely poor. This is the first report to compared the treatment results between generations of CUP and examined prognostic factors.

**Methods:**

This retrospective single‐center cohort study enrolled 68 patients with newly diagnosed unfavorable cancer of unknown primary at Taipei Veteran General Hospital from 2017 to 2020 as study cohort and 167 patients from 2000 to 2009 as historical cohort.

**Results:**

The median overall survival was 4.3 months in the study cohort (95% CI, 2.7–6.2 months) and 4.5 months in the historical cohort (95% CI, 3.0–5.5 months; *p* = 0.858). Eleven patients in the study cohort received immunotherapy. The disease control rates were 45%. Multivariate analysis showed that an Eastern Cooperative Oncology Group score > 1 and a C‐reactive protein level > 1 correlated with poor survival. A new prognostic stratification model was constructed by using Eastern Cooperative Oncology Group score and C‐reactive protein values. The good‐, intermediate‐, and poor‐risk groups had distinct median overall survival of 18.3, 7.0 and 1.2 months, respectively (area under the curve, 0.817; *p* < 0.001).

**Conclusion:**

The outcome of unfavorable cancer of unknown primary has not changed much over the last 20 years. The application of a new prognostic stratification model can further stratify unfavorable cancer of unknown primary.

## Graphical abstract

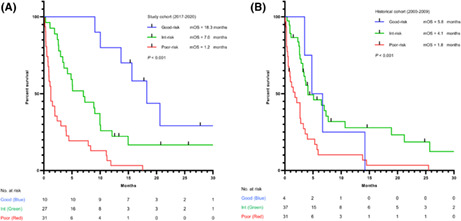

## 1 INTRODUCTION

Cancer of unknown primary (CUP) is defined as a confirmed metastatic malignancy without definite origin by complete examination. The CUP incidence is 2.72/100,000 people and accounts for 0.55% of newly diagnosed cancers annually in Taiwan.^1^ In the U.S.A., the incidence is 9.87/100,000 people, and CUP accounts for 1.7% of all cancers.^2^ The histology, clinical presentation, and prognosis of CUP are extremely heterogeneous, and the median overall survival (OS) of unfavorable CUP is poor (approximately 6–12 months).^3^ To define the origin of the primary tumor, patients need thorough examinations, including endoscopy, computed tomography, magnetic resonance imaging, and positron emission tomography. Utilization of next‐generation sequencing and bioinformatics analyses has broaden our understanding and helps to categorize CUP into specific subtypes, such as lung cancer and pancreatic cancer.^4^, ^5^, ^6^ Novel treatments such as immunotherapy and targeted therapy have been developed and investigated in clinical trials and practice.^7^, ^8^

In a previous study, we analyzed 179 unfavorable CUP patients diagnosed during 2000–2009 at Taipei Veterans General Hospital, which is a tertiary medical center in Taiwan. The median overall survival was 6.2 months. Multivariate analysis revealed several important prognostic factors, including performance status and serum albumin and calcium levels.^9^ Approximately 10 years later, we established a multidisciplinary team (MDT) for CUP in 2017. Members included medical oncologists/hematologists, pathologists, radiologists, and nuclear medicine specialists. Newly diagnosed unfavorable CUP was discussed at the MDT conference to achieve optimal management. Updated treatments, including targeted therapy and immune checkpoint inhibitors (ICIs), were also recommended based on the committee discussion. In this study, we compared the treatment results between generations of CUP and examined prognostic factors.

## 2 MATERIAL AND METHODS

This was a retrospective study conducted at Taipei Veterans General Hospital. Patients who were diagnosed with unfavorable CUP at Taipei Veterans General Hospital from December 1st, 2017, to July 30th, 2020 and discussed at the MDT conference were enrolled in the study cohort. The historical cohort included patients diagnosed from January 1 2000, to December 31, 2009, at the same hospital from chart review. Patients with pathologically confirmed malignancy were included. None of patients in study cohort and historical cohort received postmortem dissection. Favorable CUP was excluded in both cohorts if the following features were presented: Adenocarcinoma with axillary lymph node metastasis in females; squamous cell carcinoma in head and neck lymph nodes; papillary or serous carcinoma in the peritoneal cavity in females; a solitary and potentially resectable tumor; poorly differentiated carcinoma with midline nodal disease in young males; squamous cell carcinoma with isolated inguinal lymph node metastasis; adenocarcinoma with osteoblastic bone metastasis; and elevated prostate‐specific antigen in males. Neuroendocrine tumors and neuroendocrine carcinomas were included in previous study as unfavorable CUP.^9^ However, neuroendocrine tumors and carcinomas were in distinct disease entities that harbor favorable prognosis and were generally treated as another disease based on recent studies and NCCN guideline.^3^, ^10^ Thus, we excluded neuroendocrine tumors and carcinomas in this study. The diagnosis of unfavorable CUP in historical cohort was retrospectively reviewed according to the pathology, imaging, and laboratory data of patients fitted the inclusion criteria of this study. Any case with identified tumor origin or favorable features that meet the exclusion criteria would be excluded during the review process. Clinical information was collected and included patient characteristics, pathology, treatment, and blood tests. The patient performance status was assessed by the Eastern Cooperative Oncology Group score (ECOG).

Treatment was individualized based on the patient's general physical status, involved sites and assumed tumor of origin. Low‐intensity chemotherapy or supportive care was chosen if the patient presented poor performance status. For head and neck origin, chemotherapy, radiotherapy, cetuximab or immunotherapy was suggested. FOLFOX was recommended for assumed gastrointestinal origin. For symptomatic spinal metastasis or lymphadenopathy, radiotherapy was considered. In general, chemotherapy containing platinum, taxane, fluoropyrimidine, or gemcitabine was an appropriate treatment for cancer of unknown primary.^3^

Overall survival (OS) was defined as the time from pathological diagnosis until either date of death or date of last follow‐up. An independent *t* test was performed to evaluate differences in the characteristics between the study cohort and historical cohort. Subgroup analysis was subsequently performed. The Kaplan–Meier method was used to generate survival curves. Univariate analysis of overall survival was performed with the log rank test, and *p* < 0.05 (two‐tailed) was considered significant. Multivariate Cox regression analyses was subsequently performed using backward, stepwise selection to derive a multivariate model for significant predictors. Only predictors with significance were chosen as the elements of the new prognostic stratification model. The area under the curve (AUC) of the prognostic prediction model was estimated. Statistical analyses were conducted using SPSS software version 22 (SPSS Inc.). *p* < 0.05 was considered a statistically significant difference.

## 3 RESULTS

Sixty‐eight patients with unfavorable CUP were identified in the study cohort. One hundred and seventy nine patients were originally included in the historical cohort, but 12 of them were excluded due to diagnosis of neuroendocrine carcinoma. Finally, 167 patients were identified in the historical cohort. The characteristics of the two cohorts are listed in Table 1. In the study cohort, 37 of 68 (54%) patients were female, and 31 of 68 (46%) were male. The sex ratio was balanced. The median age upon diagnosis was 65 years (interquartile range [IQR], 56–79 years). Adenocarcinoma was the most common histology (38%), followed by poorly differentiated adenocarcinoma or carcinoma (35%), carcinoma not otherwise specified (NOS; 16%), squamous cell carcinoma (9%), and others (2%, one patient with angiosarcoma). CUP was diagnosed most commonly in the abdomen (69%), lung (44%), bone (31%), neck (28%), and liver (24%). Forty‐three of 68 (63%) patients had metastasis involving more than one organ. More than half of the patients (58%) had a poor performance status (ECOG ≥ 2). Twelve of 68 (18%) patients were in bed‐ridden status (ECOG = 4).

**TABLE 1:** Characteristics of unfavorable CUP in the study and historical cohorts

Characteristic	2017–2020 (*n* = 68)	2000–2009 (*n* = 167)	*p* value
Median OS, months (range)	4.3 (2.7–6.2)	4.5 (3.0–5.5)	0.858
Median age at diagnosis, years (IQR)	65 (56–79)	73 (58–79)	0.242
Sex (%)			
Male	31 (46)	118 (71)	<0.001
Female	37 (54)	49 (29)	
Histology (%)			
Adenocarcinoma	26 (38)	63 (38)	0.942
Carcinoma NOS	11 (16)	63 (38)	0.001
Poorly differentiated carcinoma/adenocarcinoma	24 (35)	28 (17)	0.002
Squamous cell carcinoma	6 (9)	6 (3)	0.099
Others	1 (2)	7 (4)	0.518
ECOG (%)			
0–1	29 (42)	75 (45)	0.751
2–4	39 (58)	92 (55)	
Metastasis sites (%)			
Neck	19 (28)	36 (22)	0.295
Lung	30 (44)	86 (51)	0.305
Abdomen	47 (69)	31 (19)	<0.001
Liver	16 (24)	74 (44)	0.003
Bone	21 (31)	68 (41)	0.159
Brain	6 (9)	14 (8)	0.913
Axillary or inguinal lymph node	5 (7)	14 (8)	0.793
Numbers of metastatic sites (%)			
1	25 (37)	38 (23)	0.028
≥2	43 (63)	129 (77)	
Treatment (%)			
Best supportive care	21 (31)	44 (26)	0.483
Chemotherapy	43 (63)	108 (65)	0.836
Radiotherapy	16 (24)	48 (29)	0.418
Target therapy	20 (29)	8 (5)	<0.001
Immunotherapy	11 (16)	0 (0)	<0.001
Chemotherapy regimen (%)			
Platinum‐based	27 (63)	84 (78)	0.140
Fluoropyrimidine based	29 (67)	44 (41)	0.021
Etoposide‐based	2 (5)	43 (40)	<0.001
Gemcitabine‐based	8 (19)	27 (25)	0.367
Taxane‐based	20 (47)	21 (19)	0.007
Oxaliplatin‐based	9 (21)	14 (13)	0.301

**FIGURE 1:**
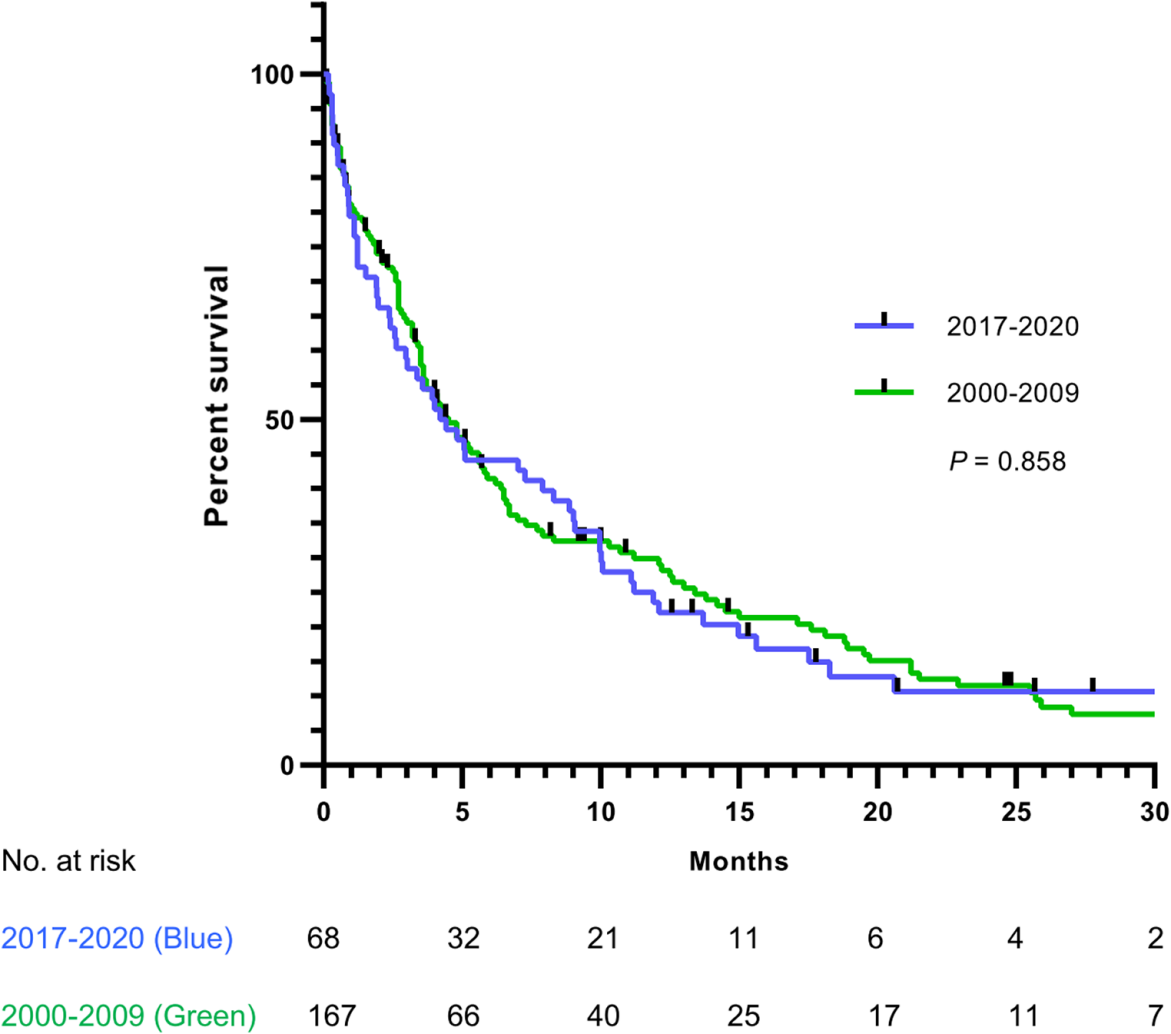
Overall survival of the study and historical cohorts

The median OS in the study cohort was 4.3 months (95% CI: 2.7–6.2 months), which was similar to that in the historical cohort after excluding neuroendocrine carcinoma (median OS, 4.5 months; 95% CI: 3.0–5.5 months; *p* = 0.858). The survival curve is shown in Figure 1. Supportive care was adopted in 31% of patients in the study cohort, while others received palliative treatment, including chemotherapy (63%), radiotherapy (24%), target therapy (29%), and immune checkpoint inhibitors (16%). The most commonly used chemotherapy was fluoropyrimidine (67%), followed by platinum (63%), taxane (47%), oxaliplatin (21%), gemcitabine (19%), and etoposide (5%).

In the historical cohort, males were predominant (male, 71%; female, 29%; *p* < 0.001), and the patients were older than those in the study cohort (median age upon diagnosis, 73 years; IQR, 58–79 years; *p* = 0.242). Adenocarcinoma and carcinoma NOS were the most common histology subtypes, each comprising 38% of the population. Other CUP patients were diagnosed with poorly differentiated adenocarcinoma or carcinoma (17%), squamous cell carcinoma (3%) or others (4%; 5 patients with sarcoma and 2 patients with melanoma). A total of 129 of 167 (77%) patients had metastasis at more than one organ. Fifty‐five percent of patients had ECOG ≥ 2, and 12% of patients were in bed‐ridden status.

Chemotherapy was adopted in 65% patients. Fewer patients received targeted therapy (5%; *p* < 0.001), and no patients received immune checkpoint inhibitors. The most commonly used chemotherapy was platinum (78%), followed by fluoropyrimidine (41%), and etoposide (40%). There were markedly fewer patients receiving taxane (19%; *p* = 0.007).

Among the study cohort, 11 patients received immune checkpoint inhibitors (ICIs). Eight of these patients received pembrolizumab, four received nivolumab, and two received atezolizumab. Three of these patients received anti‐vascular endothelial growth factor target therapy and ICI concomitantly. The resected or biopsied tissue was sent for programmed cell death ligand 1 (PD‐L1) expression analysis by immunochemical test 22C3 pharmDx assay (Agilent/Dako) or 28‐8 pharmDx assay (Agilent/Dako). Five of these patients had positive PD‐L1 expression. Responses were observed in five patients, including four patients with partial response and one patient with stable disease. The disease control rate was 45%. The median progression‐free survival (PFS) was 2.3 months (95% CI: 0–6.47 months). The median OS was 10.03 months (95% CI: 3.42–16.65 months). The duration of ICI treatment ranged from 8 to 12 months in the responding patients. The detailed characteristics of the ICI group are shown in Table 2.

**TABLE 2:** Unfavorable CUP patients receiving immunotherapy in the study cohort

Age/Sex	Histology	PD‐L1 stain	Cancer treatment	ICI response	PFS, months	OS, months
50/Female	Poorly differentiated adenocarcinoma	Not done^a^	Pembrolizumab + afatinib × 1 cycle, atezolizumab + TS‐1 × 1 year	PR	23.2	25.7
61/Male	Squamous cell carcinoma	TPS:20% CPS:20% TC:15%	TPF, CCRT, pembrolizumab × 9 months, CCRT, paclitaxel × 2 cycles	SD	10.4	15.6
59/Male	Squamous cell carcinoma	TPS:1% CPS:10% TC: 0.8%	TPF + pembrolizumab × 2 cycles, pembrolizumab + sorafenib + afatinib × 1 cycles, pembrolizumab + sorafenib + afatinib × 2 cycles, cetuximab × 6 cycles, atezolizumab × 1 cycle	PR	5	11.9
52/Male	Poorly differentiated carcinoma	TC:70%	CCRT, PFL × 2 cycles, paclitaxel + ifosfamide × 1 cycle, MEMOCLUB × 1 cycle, nivolumab × 2 cycles	PD	0.5	11.2
48/Male	Carcinoma, NOS	CPS:1%	CCRT, paclitaxel + cisplatin × 6 months, RT, everolimus, pembrolizumab × 1 cycle	PD	0.8	10.0
51/Male	Squamous cell carcinoma	TC: 0%	Pembrolizumab × 5 months, pembrolizumab + PFL × 2 cycles, pembrolizumab + cetuximab × 1 cycle, pembrolizumab + cetuximab + afatinib × 1 cycle	PR	4.6	8.9
53/Female	Adenocarcinoma	Not done^a^	PFL × 2 cylces, Olaparib × 1 cycle, pembrolizumab × 1 cycle	Undetermined	0.1	5.1
64/Female	Carcinoma, NOS	TPS:50% CPS:50%	Paclitaxel + carboplatin × 2 cycles, bavacizumab + GC × 2 cycles, pembrolizumab + GC × 2 cycles	PD	1.1	4.4
53/Female	Poorly differentiated carcinoma	TC: 0%	Surgery, CCRT, PFL × 6 cycles, nivolumab + PFL × 8 months	PR	8.4	15.3
50/Female	Carcinoma, NOS	TC: 0%	Nivolumab × 2 months, pembrolizumab + gemcitabine + paclitaxel × 1 cycle	PD	2.3	2.6
51/Male	Squamous cell carcinoma	Not done^a^	PFL × 1 cycle, gemcitabine + oxaliplatin + cetuximab + nivolumab × 1 cycle	PD	0.4	0.5

Abbreviations: CCRT, concurrent chemoradiotherapy; CPS, combined positive score; CUP, cancer of unknown primary; GC, gemcitabine, cisplatin; MEMOCLUB, methotrexate, epirubicin, mitomycin‐C, vincristine, cisplatin, leucovorin, 5‐fluorouracil, bleomycin; OS, overall survival; PD, progressive disease; PD‐L1, programmed death‐ligand 1; PFL, cisplatin, 5‐fluorouracil, leucovorin; PFS, progression free survival; PR, partial response; RT, radiotherapy; SD, stable disease; TC, tumor cells; TPF, docetaxel, cisplatin, 5‐fluorouracil; TPS, tumor proportion score.

^a^ Inadequate sample for PD‐L1 staining.

Twenty patients received target therapy in study cohort. Commonly used drugs were bevacizumab (40%), cetuximab (15%), and afatinib (20%). Most target therapy (74%) was used in combination with chemotherapy or immunotherapy. The disease control rate was 60% including one complete response, five partial response, and eight stable disease. The median PFS was 2.9 months (95% CI: 0.86–4.95 months). The median OS was 9.97 months (95% CI: 7.85–12.09 months). Other details are described in Table S1.

Univariate analysis of the study cohort was performed to identify prognostic factors. The results are shown in Table 3. Important characteristics that correlated with poor prognosis included age > 65 years (median OS, 2.97 months; *p* = 0.053), male sex (median OS, 2.57 months; *p* = 0.003), ECOG ≥ 2 (median OS, 1.93 months; *p* < 0.001), the presence of lung metastasis (median OS, 3.93 months; *p* = 0.03) and liver metastasis (median OS, 1.93 months; *p* = 0.017). In the study cohort, 93% of patients with ECOG scores of 0–1 received at least one cancer treatment, including surgery, radiotherapy, chemotherapy, targeted therapy or immunotherapy. However, only 51% of patients with ECOG scores ≥ 2 were treated. The impact of ECOG in the treatment group was significant, and the median OS was 10.1 and 3.6 months for ECOG scores of 0–1 and ≥2, respectively (*p* = 0.011). Although the outcome was poor for patients with ECOG scores ≥ 2, the benefit of receiving any one cancer treatment still demonstrated that the median OS increased from 1.1 months to 3.6 months (*p* = 0.001). Overall, patients who were not amenable to receiving any anticancer therapy had much shorter survival than the treatment group, with a median OS of 1.1 months versus 9.0 months (*p* < 0.001). Patients receiving targeted therapy, radiotherapy or immunotherapy had better survival, although these differences were not statistically significant (median OS for targeted therapy, 9.9 months; *p* = 0.065; median OS for radiotherapy, 10.0 months; *p* = 0.089; median OS for immunotherapy, 10.0 months; *p* = 0.244).

**TABLE 3:** Univariate and multivariate analysis of prognostic factors (Study cohort: 2017–2020)

Variable	Number	Univariate		Multivariate	
		Median OS (months)	*p* value	HR (95% CI)	*p* value
Age (years)					
≥65	35	2.97	0.053		
<65	33	9.03			
Sex					
Male	31	2.57	0.003	1.23 (0.62–2.43)	0.552
Female	37	9.03		1 (Reference)	
ECOG					
2–4	39	1.93	<0.001	2.37 (1.30–4.31)	0.005
0–1	29	10.03		1 (Reference)	
BMI					
<24	31	4.83	0.670		
≥24	37	4.20			
Smoking					
Yes	28	3.37	0.157		
No	40	4.83			
Histology					
Adenocarcinoma	26	7.27	0.269		
Carcinoma, NOS	11	2.57			
Poorly differentiated adeno/carcinoma	24	4.2			
SCC	6	1.23			
Others	1	1.9			
Lung involvement					
Yes	30	3.93	0.032	1.10 (0.58–2.07)	0.773
No	38	7.27		1 (Reference)	
Liver involvement					
Yes	16	1.93	0.017	1.87 (0.99–3.55)	0.054
No	52	7.03		1 (Reference)	
Brain involvement					
Yes	6	7.9	0.955		
No	62	4			
No of metastatic site					
≥3	27	4.0	0.262		
1–2	41	5.10			
Chemotherapy					
Yes	43	9.03	<0.001		
No	25	1.10			
Radiotherapy					
Yes	16	10.0	0.089		
No	52	2.97			
Targeted therapy					
Yes	20	9.97	0.065		
No	48	2.97			
Immunotherapy					
Yes	11	10.03	0.244		
No	57	3.47			
WBC count (mm^3^)					
≥10,000	23	1.53	<0.001		
<10,000	45	9.03			
Hgb level (g/dl)					
≥11	41	7.03	0.534		
<11	27	3.57			
Platelet count (mm^3^)					
≥150,000	60	4.43	0.663		
<150,000	8	2.97			
ANC (mm^3^)					
≥4000	55	3.93	0.214		
<4000	13	9.07			
NLR					
≥4	47	2.63	0.011	1.30 (0.65–2.58)	0.459
<4	21	10.03		1 (Reference)	
Alb level (g/dl)					
≥3.5	39	9.07	<0.001	0.62 (0.34–1.14)	0.123
<3.5	29	2.4		1 (Reference)	
LDH level (IU)					
≥250	38	3.37	0.254		
<250	30	7.03			
Na level (mmol/L)					
≥135	50	8.3	<0.001	0.66 (0.32–1.38)	0.271
<135	18	1.93		1 (Reference)	
CRP level (mg/dl)					
≥1	50	2.97	<0.001	2.05 (1.02–4.14)	0.044
<1	18	13.7		1 (Reference)	

Abbreviations: Alb, albumin; ANC, absolute neutrophil count; BMI, body mass index; CRP, C‐reactive protein; Hgb, hemoglobin; LDH, lactate dehydrogenase; Na, sodium; NLR, neutrophil‐to‐lymphocyte ratio; SCC, squamous cell carcinoma; WBC, white blood cell.

Laboratory parameters implicating poor prognosis included white blood cell count (WBC) ≥ 10,000/mm^3^ (median OS, 1.53 months; *p* < 0.001), neutrophil‐to‐lymphocyte ratio (NLR) ≥ 4 (median OS, 2.63 months; *p* = 0.011), serum albumin level <3.5 g/dl (median OS, 2.4 months; *p* < 0.001), serum sodium level < 135 mmoL/L (median OS, 1.93 months; *p* < 0.001), and CRP level ≥ 1 mg/dl (median OS, 2.97 months; *p* < 0.001). The multivariate analysis confirmed sustained significance of the performance status (hazard ratio [HR], 2.37; *p* = 0.005) and CRP level (HR, 2.05; *p* = 0.044).

**FIGURE 2:**
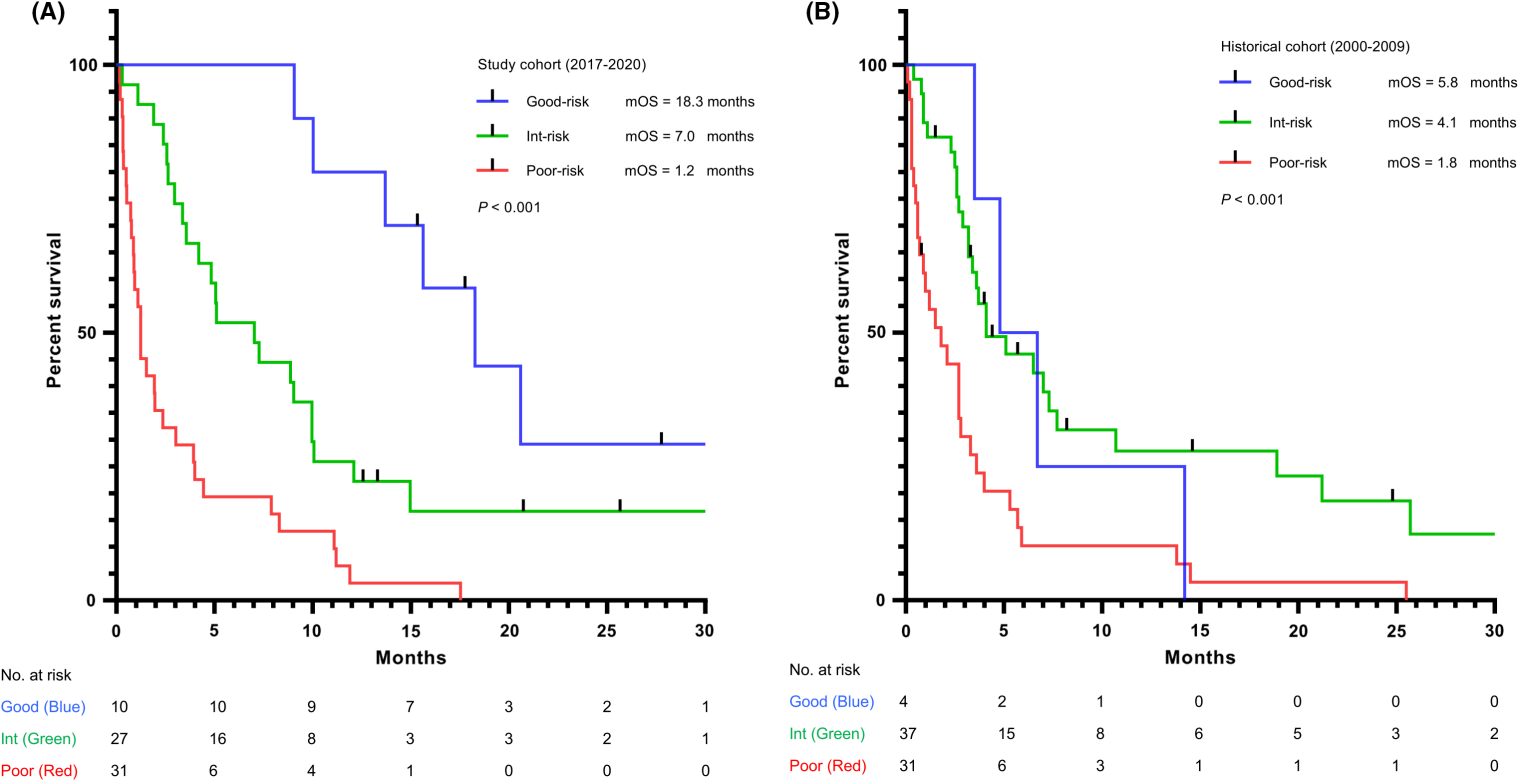
Overall survival (part a) of the study cohort according to the unfavorable CUP prognostic stratification model and validation with historical cohort (part b)

We proposed a new prognostic stratification model for unfavorable CUP. Patients were assigned to three different risk groups based on two parameters, ECOG and CRP (ECOG 0–1 and CRP <1 mg/dl, good‐risk; ECOG 2–4 or CRP ≥ 1 mg/dl, intermediate‐risk; ECOG 2–4 and CRP ≥ 1 mg/dl, poor‐risk). The median OS of the good‐, intermediate‐ and poor‐risk groups was 18.3, 7.0, and 1.2 months, respectively (*p* < 0.001), and the survival curve is shown in Figure 2. The area under the curve (AUC) was 0.817. We also validated the prognostic stratification model in the historical cohort. In this cohort, 72/167 (43%) patients had CRP data upon diagnosis. The median OS of the good‐, intermediate‐ and poor‐risk groups was 5.8, 4.1, and 1.8 months, respectively (*p* < 0.001), and the AUC was 0.613.

## 4 DISCUSSION

This study compared the change in epidemiology, characteristics, treatment, and prognosis of unfavorable CUP in two generations of patients at a single site. Although the outcome of unfavorable CUP remained poor and the median OS was only 4 months, many aspects of unfavorable CUP have changed after a decade.

In the current cohort, patients with unfavorable CUP were diagnosed younger, and the ratio of males to females was equivalent. This reflected that after the multimodality approach, the characteristics of the unfavorable CUP population became more similar to those of the general population, according to the Taiwan cancer registry reports and reports from Western countries.^1^, ^11^ In Carmen Binder's report between 1981 and 2014, 47% of CUP patients were male, and the mean diagnosis age was 72 years in the Cancer Registry of Zurich.^12^ In another report conducted between 2014 and 2016 in Memorial Sloan Kettering Cancer Center, 53% of CUP patients were male, and the mean diagnosis age was 65 years.^13^ The immunochemical test in CUP diagnosis has also improved in recent decades. Experienced pathologists use morphology, screening markers and staining for lineage‐restricted transcription factors to determine the cancer cell type and origin. A structural pathologic diagnostic algorithm was proposed by Andrew M Bellizzi and was one of the references used by pathologists facing CUP.^14^, ^15^

Platinum was still the main chemotherapy agent used for the treatment of CUP. The use of fluoropyrimidine and taxane increased in these 10 years. Fluoropyrimidine is widely used in head and neck cancer, esophageal cancer, gastric cancer, pancreatobiliary cancer, and colon cancer.^16^, ^17^ A previous meta‐analysis indicated favorable outcomes with the use of platinum and taxane. The hazard ratios favored the use of platinum, taxane, or both (HR: 0.69, 0.66, and 0.81, respectively) compared to other chemotherapy agents.^18^ Thus, the preferred chemotherapy regimens were either platinum plus taxane or fluoropyrimidine‐based regimens in the updated National Comprehensive Cancer Network guidelines.^3^

Some patients received targeted therapy and immune checkpoint inhibitors in the study cohort. Nearly 30% of patients received targeted therapy, such as bevacizumab, ramucirumab, erlotinib, afatinib, lenvatinib, sorafenib, and cetuximab. Many of these medications have been developed and available for clinical use in the past 10–15 years. Each targeted therapy had distinct use in specific cancer types. Bevacizumab, ramucirumab, and cetuximab were approved for colon cancer treatment.^19^, ^20^, ^21^ Erlotinib and afatinib were incorporated in lung cancer treatment.^22^, ^23^ Sorafenib and lenvatinib were used for renal cell carcinoma, hepatocellular carcinoma and thyroid cancer.^24^, ^25^, ^26^, ^27^, ^28^, ^29^

Immune checkpoint inhibitors, such as pembrolizumab, nivolumab or atezolizumab, were developed very recently. However, their use has been rapidly increasing in both clinical trials and in the real world due to potentially robust responses. A Phase 2, open‐label trial of pembrolizumab in refractory CUP demonstrated a 23% overall response.^7^ Another phase 2 trial of nivolumab in CUP also produced a 24% overall response in previously treated patients.^8^ The median progression‐free survival was approximately 5 months, but the median overall survival approached 15 months. The response was even higher in CUP patients with high PD‐L1 expression. In our study cohort, ICIs were chosen as cancer treatment in 11 patients based on either the evidence of high PD‐L1 expression or physician experience. Disease control rates were 45%, and ICI treatment remained for at least 8 months. The outcome was remarkable. Therefore, checking the PDL1 status of unfavorable CUP patients and providing anti‐PD1 therapy seems to be a new alternative treatment.

Several poor prognostic factors, such as poor performance status, older age, number of metastatic organs, high serum lactate dehydrogenase (LDH) level, high serum CRP level, low serum albumin level, leukocytosis, high NLR, and visceral metastasis, were commonly mentioned in previous studies.^30^, ^31^, ^32^, ^33^, ^34^, ^35^, ^36^ In the current study, the above clinical parameters were tested, but only the performance status and serum CRP level remained significant after multivariate analyses. The baseline performance status reflected the composite effect of aging, underlying disease, cancer‐associated fatigue, depression, anorexia, malnutrition, inflammation, and infection. One of the assessment tools, ECOG, was used universally in the clinical practice setting to define patient fitness for cancer treatment. Stéphane Culine proposed a prognostic model using performance status, LDH level and liver metastasis to stratify CUP patients into good‐ or poor‐risk groups.^33^ The median OS of the good‐risk group was 11.7 months, and that of the poor‐risk group was 3.9 months (*p* < 0.001). We validated Culine's prognostic model with our study cohort. The median OS of good‐risk vs. poor‐risk group was 15.6 versus 3.0 months (*p* < 0.001). Although the patients could be stratified into different risk group, 84% patients in our cohort were in poor‐risk group. The characteristic difference between Culine's cohort and our cohort might be the reason. The median OS of Culine's cohort was 7.5 months versus 4.3 months in our cohort. Poor ECOG group composed 33% population in Culine's cohort and 58% population in our cohort. High‐LDH group composed 22% population in Culine's cohort and 56% population in our cohort. Considering more poor‐prognosis patients in our cohort, we supposed our new prognostic model is more suitable for unfavorable CUP.

CRP is an acute phase reactant that is indicative of cancer‐related inflammation and associated with advanced disease and aggressiveness.^37^ One of the inflammation‐based scores, the so‐called modified Glasgow prognostic score, incorporated serum CRP (≥10 mg/dl), and albumin (≥3.5 g/L) levels to predict the prognosis of unfavorable CUP in a previous study (HR, 1.53; *p* = 0.03).^35^

Dimitrios et al. identified three prognostic factors, performance status, leukocytosis and visceral metastasis, and developed the Ioannina Score for CUP Outpatient Oncologic Prognostication by Classification and Regression Tree analysis.^30^ Along with the first step in the algorithm, physicians should allocate CUP into three subgroups based on clinicopathological features. The low‐risk group was scored 0 points and comprised serous papillary peritoneal carcinomatosis, axillary nodal disease and squamous cell carcinoma at the head and neck region. The intermediate‐risk group was scored 1 point and comprised nodal disease, neuroendocrine tumor and mucinous peritoneal carcinomatosis. The others were scored 2 and went through the algorithm to plus another 1 or 2 points if leukocytosis was present (WBC ≥ 10,000/mm^3^), ECOG ≥2 or both. The median survival of the 0‐, 1‐, 2‐, 3‐, and 4‐point groups was 36, 14, 11, 8, and 5 months, respectively.

In this study, we focused on the prognostic analysis of the unfavorable subset of CUP. Although the serum albumin level was considered to be a prognostic factor in the historical cohort, it was not found to be a significant prognostic factor in the study cohort by multivariate analysis (HR, 0.62; *p* = 0.123). Thus, the albumin‐derived inflammation‐based parameter, modified Glasgow prognostic score, was not incorporated into model building. A simplified grouping method was applied instead of a complicated mathematical method for convenient clinical utilization. The prognostic prediction model using ECOG and CRP could clearly stratify patients into totally different prognostic groups. We also utilized a historical cohort to validate the prognostic stratification model. However, the survival difference between the good‐risk and intermediate‐risk groups was smaller. The AUC also showed a lower predictive value for the prognostic stratification model in the historical cohort. Because CRP was frequently tested in situations of fever, illness and infection, a large proportion of patients presenting as afebrile and in good health would not receive CRP testing. Overall, 72 of the 167 patients (43%) in the historical cohort had CRP data upon diagnosis, but only four patients were in the good‐risk group. This high rate of missing data and uneven distribution resulted in selection bias of the historical cohort.

A limitation of this study was the inevitable retrospective analysis and more selective bias, especially from the historical cohort. In addition, none of patients in study cohort and historical cohort received postmortem dissection. Also, the assessment of ECOG was relatively subjective. The difference between ECOG 1 and 2 and between ECOG 2 and 3 depended partially on the physician's opinion. However, this prognostic stratification model could still provide physicians with a simple method to determine prognosis before treating a patient with unfavorable CUP.

## 5 CONCLUSION

The diagnosis and treatment of unfavorable CUP have changed dynamically during these 20 years. Such changes include an increase in the use of second‐generation chemotherapy and the introduction of targets and immunotherapy. However, the outcomes of CUP remained poor. Performance status and inflammation parameters were still the most important prognostic factors. A new prognostic prediction model is proposed and can further facilitate the prediction of prognosis.

## AUTHOR CONTRIBUTIONS

Dr Chang had full access to all of the data in the study and takes responsibility for the integrity of the data and the accuracy of the data analysis. Concept and design: Tang, Chang, Hang, Hung, Lai, Chen. Acquisition, analysis, or interpretation of data: All authors. Drafting of the manuscript: Tang, Chang. Statistical analysis: Tang, Chang. Supervision: Chang.

## CONFLICT OF INTEREST

The authors have no conflict of interest.

## ETHICS STATEMENT

This study was reviewed and approved by the Taipei Veterans General Hospital Institutional Review Board and Ethics Committee (IRB No.:2021‐02‐009CC).

## Supporting information


Table S1

## Data Availability

The data that support the findings of this study are available on request from the corresponding author. The data are not publicly available due to privacy or ethical restrictions.
