# Supplementary material for: A generational comparison for unfavorable cancer of unknown primary in a single institute over 20 years
Source: Cancer Med. 2022 Jul 3;12(2):1090–101. doi: 10.1002/cam4.4960 (PMC9883408; doi:10.1002/cam4.4960)
Supplement: Supplementary file 1 — Table S1 [file CAM4-12-1090-s001.docx]

Supplement. Table S1. Unfavorable CUP patients receiving target therapy in the study cohort

| Target therapy | Class | Number | Regimen (n) | Response (n) | PFS, months  (Range) | OS, months  (Range) |
| --- | --- | --- | --- | --- | --- | --- |
| Bevacizumab  (Bev) | Anti-VEGF mAb | 8 | Bev + FOLFOX (3), Bev + FOLFIRI (1), Bev + paclitaxel (1), Bev + cisplatin + paclitaxel (1), Bev + gemcitabine + carboplatin (1),  Bev + gemcitabine + cisplatin (1) | CR (1), PR (1), SD (6) | 2.2-45 | 4.4-47.5 |
| Ramucirumab | Anti-VEGFR mAb | 1 | Ramucirumab + cisplatin + docetaxel (1) | PD (1) | 2.9 | 13.7 |
| Cetuximab | Anti-EGFR mAb | 3 | Cetuximab (1), Cetuximab + afatinib + pembrolizumab (1), Cetuximab + gemcitabine + oxaliplatin + nivolumab (1) | PR (1), PD (1), Undetermined (1) | 0.4-4 | 0.5-11.9 |
| Erlotinib | Anti-EGFR TKI | 2 | Erlotinib + gemcitabine + paclitaxel (1), Erlotinib + FOLFIRINOX (1) | Undetermined (2) | 0.1-0.6 | 0.9-3.0 |
| Afatinib | MTKI | 4 | Afatinib (1), Afatinib + sorafenib + pembrolizumab (1), Afatinib + cetuximab + pembrolizumab (1),  Afatinib + pembrolizumab (1) | PR (2), SD (1), PD (1) | 0.6-3.3 | 0.9-25.7 |
| Lenvatinib | MTKI | 2 | Lenvatinib (2) | PR (1), SD (1) | 3.5-11.2 | 12.5-20.6 |
| Sorafenib | MTKI | 1 | Sorafenib + afatinib + pembrolizumab (1) | PR (1) | 3.3 | 11.9 |
| Everolimus | mTOR inhibitor | 1 | Everolimus (1) | PD (1) | 1.3 | 10.0 |
| Olaparib | PARP inhibitor | 1 | Olaparib (1) | PD (1) | 1.6 | 5.1 |

Abbreviations: CUP, cancer of unknown primary; PFS, progression free survival; OS, overall survival; CR, complete response; PR, partial response; SD, stable disease; PD, progressive disease; VEGF, vascular endothelial growth factor; EGFR, epidermal growth factor receptor; mTOR, mammalian target of rapamycin; PARP, poly (ADP-ribose) polymerase; mAb, monoclonal antibody; TKI, tyrosine kinase inhibitor; MTKI, multi-tyrosine kinase inhibitor; FOLFOX, 5-fluorouracil, leucovorin, oxaliplatin; FOLFIRI, 5-fluorouracil, leucovorin, irinotecan; FOLFIRINOX, 5-fluorouracil, leucovorin, irinotecan, oxaliplatin
